# Mitochondrial Genome Variants as a Cause of Mitochondrial Cardiomyopathy

**DOI:** 10.3390/cells11182835

**Published:** 2022-09-11

**Authors:** Teresa Campbell, Jesse Slone, Taosheng Huang

**Affiliations:** Department of Pediatrics, Jacobs School of Medicine and Biomedical Sciences, University at Buffalo, Buffalo, NY 14203, USA

**Keywords:** mtDNA, mitochondrial genome, mitochondrial cardiomyopathy, hypertrophic cardiomyopathy, dilated cardiomyopathy, reactive oxygen species, calcium, iron overload, ferroptosis

## Abstract

Mitochondria are small double-membraned organelles responsible for the generation of energy used in the body in the form of ATP. Mitochondria are unique in that they contain their own circular mitochondrial genome termed mtDNA. mtDNA codes for 37 genes, and together with the nuclear genome (nDNA), dictate mitochondrial structure and function. Not surprisingly, pathogenic variants in the mtDNA or nDNA can result in mitochondrial disease. Mitochondrial disease primarily impacts tissues with high energy demands, including the heart. Mitochondrial cardiomyopathy is characterized by the abnormal structure or function of the myocardium secondary to genetic defects in either the nDNA or mtDNA. Mitochondrial cardiomyopathy can be isolated or part of a syndromic mitochondrial disease. Common manifestations of mitochondrial cardiomyopathy are a phenocopy of hypertrophic cardiomyopathy, dilated cardiomyopathy, and cardiac conduction defects. The underlying pathophysiology of mitochondrial cardiomyopathy is complex and likely involves multiple abnormal processes in the cell, stemming from deficient oxidative phosphorylation and ATP depletion. Possible pathophysiology includes the activation of alternative metabolic pathways, the accumulation of reactive oxygen species, dysfunctional mitochondrial dynamics, abnormal calcium homeostasis, and mitochondrial iron overload. Here, we highlight the clinical assessment of mtDNA-related mitochondrial cardiomyopathy and offer a novel hypothesis of a possible integrated, multivariable pathophysiology of disease.

## 1. Introduction

### 1.1. Mitochondria: “The Powerhouse of the Cell”

**Figure 1:**
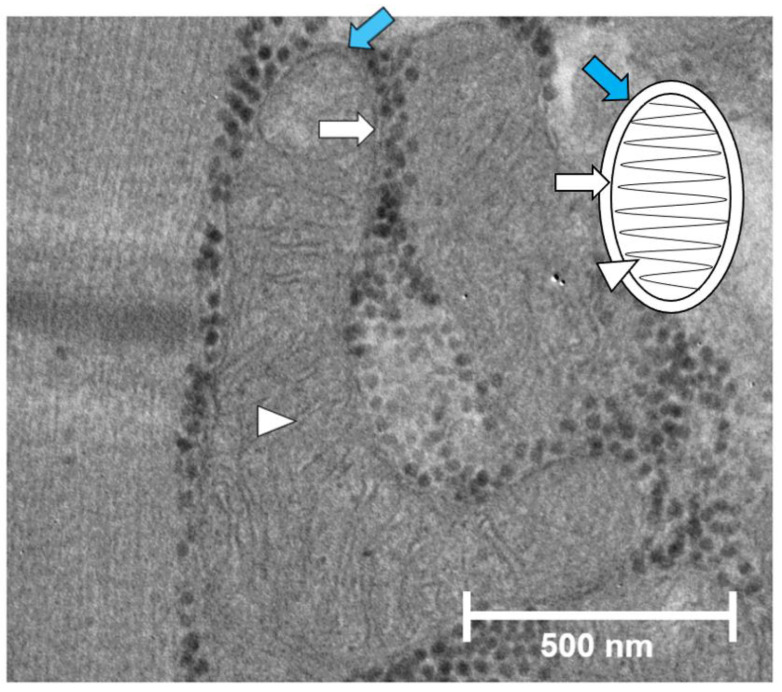
Structure of the IMM and OMM in muscle tissue. Blue arrow, OMM; white arrow, IMM; white arrowhead, cristae structure. Image property of the Huang Laboratory.

Mitochondria are small, double-membraned organelles present in the cells of all eukaryotic organisms. Mitochondrial membranes are made up of the outer mitochondrial membrane (OMM) and the inner mitochondrial membrane (IMM), creating two distinct compartments termed the intermembrane space and the mitochondrial matrix (Figure 1). The OMM is reminiscent of cell membranes and is impermeable to large molecules and proteins, while small molecules and ions can pass freely [1]. The IMM has a unique structure, which shares characteristics with bacterial cell membranes. The IMM is highly impermeable, requiring special transport for nearly all large proteins, small molecules, and ions [1]. Mitochondria have several roles in the cell, but the primary role is to generate energy in the form of ATP via oxidative phosphorylation (OXPHOS). ATP production can be broken down into several steps, the first of which is the production of acetyl-CoA from carbon substrates, including glucose, fatty acids, and proteins. Acetyl-CoA can then enter the tricarboxylic acid (TCA) cycle and is fully oxidized into CO_2_. During this process, the reducing equivalents NADH and FADH_2_ are generated, which are shuttled into the electron transport chain (ETC). The ETC is a series of four transmembrane complexes located in the IMM. The ETC performs a sequence of redox reactions by accepting elections from NADH and FADH_2,_ which then get passed along the chain to successive complexes with lower energy states. O_2_ acts as the final electron acceptor. As the electrons move along the complexes, the energy produced from the redox reactions pump protons via complex I, III, and IV, from the mitochondrial matrix to the intermembrane space, creating a proton gradient across the IMM. This proton motive force powers ATP synthase, also termed complex V, to produce ATP from ADP [2]. As O_2_ is required as the final electron acceptor in the ETC, mitochondria are considered O_2_-rich organelles.

A natural byproduct of mitochondrial respiration are free radicals, the majority of which are reactive oxygen species (ROS). The predominant ROS in the mitochondria are superoxide anions (O_2_^−^), which are produced by the leakage of electrons from the ETC (generally from complex I and III), which can then react with O_2_. Mitochondria compensate for ROS production by harboring several antioxidant systems to help maintain a healthy level of ROS in the organelle. O_2_^−^ production is countered by antioxidants termed superoxide dismutase, their importance highlighted by the loss or dysfunction having detrimental outcomes for the cell [3]. Besides superoxide dismutase, other antioxidant systems have emerged as important for ROS maintenance in the cell, one such example is glutathione peroxidases, a group of selenoproteins that catalyze varying reactions including the reduction of H_2_O_2_ and lipid hydroperoxides to water and alcohol derivatives, respectively [4]. This group of antioxidants have been demonstrated to play an integral role in preventing membrane lipid peroxidation, and inactivation of glutathione peroxidases can lead to cell death via ferroptosis [5]. Overall, while some level of ROS is part of normal functioning in the mitochondria, increased ROS can lead to mitochondrial and/or cell damage and subsequent disease.

### 1.2. Mitochondrial Genetics

**Figure 2:**
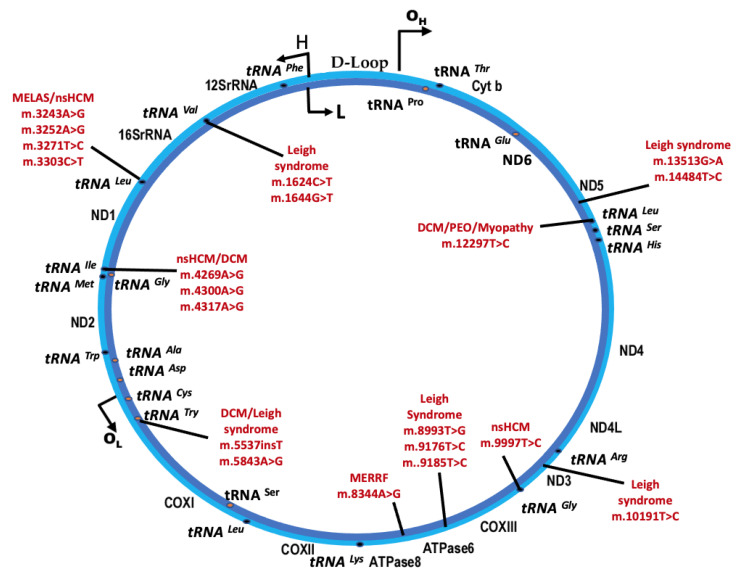
Location of select common pathogenic mitochondrial genome variants in syndromic and non-syndromic mitochondrial cardiomyopathy. H, heavy strand; L, light strand; dark ovals, tRNA location on heavy strand; orange ovals, tRNA location on light strand; tRNA, transfer RNA; DCM, dilated cardiomyopathy; nsHCM, non-sarcomeric-related hypertrophic cardiomyopathy; MELAS, mitochondrial encephalopathy, lactic acidosis, and stroke-like episodes; MERRF, myoclonic epilepsy with ragged-red fibers; PEO, progressive external ophthalmoplegia.

**Figure 3:**
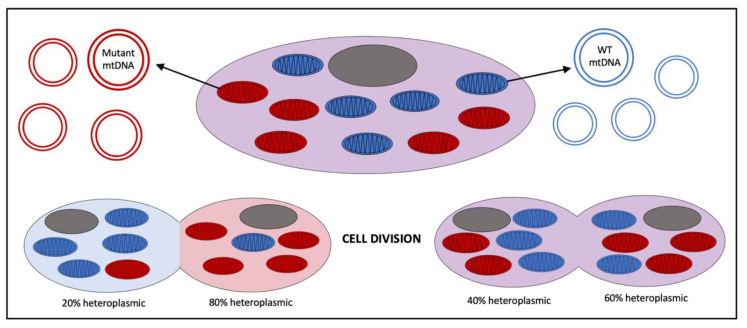
Schematic of theoretical random segregation of mitochondria during cell division and possible heteroplasmy outcomes. mtDNA, mitochondrial genome; WT, wildtype; gray oval, nucleus; blue oval, mitochondria with WT mtDNA; red oval, mitochondria with mutant mtDNA.

Mitochondria are unique from other organelles in that they contain their own circular, double-stranded DNA, mtDNA (Figure 2). While the vast majority of proteins utilized by the mitochondria (>1100) are encoded by genes in nDNA, 37 essential genes required for mitochondrial function are encoded by mtDNA [6]. Thirteen of these genes code for ETC complex proteins, twenty-two code for transfer RNAs (tRNAs), and two code for ribosomal RNAs (rRNAs). Unlike the nDNA, the mtDNA is inherited solely from the maternal germline. Each cell can contain upwards of 500–100,000 copies of mtDNA, which varies depending on the tissue type and health of the cell [7]. Deficiencies in the mtDNA copy number have been associated with multiple human diseases including cardiovascular disease, where a lower mtDNA copy number has been consistently associated with the occurrence and severity of cardiovascular disease across multiple studies [8,9]. During mitochondrial division (termed fission, to be described below), mtDNA is partitioned into the two resulting mitochondria. Like the nDNA, the mtDNA is susceptible to genetic defects, termed pathogenic variants, which can result in acquired or inherited disease. If an acquired or inherited pathogenic variant in the mtDNA is present, it can be propagated to successive mitochondria. Similarly, on the cellular level, the propagation of pathogenic mtDNA occurs through cell division. During cell division, mitochondria randomly segregate to opposite poles, creating a random pool of mitochondria in the daughter cells. Resulting cells can therefore harbor a population of mitochondria with pathogenic mtDNA, in addition to a population of mitochondria with wildtype mtDNA. This phenomenon is termed heteroplasmy. Interestingly, heteroplasmy can shift between generations [10]. As such, a mother with low heteroplasmy levels can bear children with high heteroplasmy or complete homoplasmy in certain tissues (Figure 3). Beyond inherited mtDNA variants from the maternal germline, recent attention has been placed on acquired mtDNA variants that occur with age. As the mtDNA is not partitioned from mitochondrial matrix contents (such as the nDNA in the nucleus) mtDNA molecules are more susceptible to damage from ROS within the matrix. Additionally, the DNA polymerase responsible for the replication of mtDNA, polymerase gamma (or “POLG”), has an intrinsically high error rate itself relative to the family B DNA polymerases that are used to replicate nDNA, another possible source of acquired mutations [11]. For these reasons, an increasing number of acquired mtDNA variants can compound over time, contributing to the manifestation of age-related diseases [12,13,14]. Therefore, in the healthy cell, mechanisms for the quality control and destruction of damaged mitochondria are important for cell health and function.

### 1.3. Mitochondrial Biodynamics

Despite what is often pictured in schematics or textbooks, mitochondria are dynamic organelles. They exist in fluctuating populations and the total number of mitochondria per cell may have magnitudes of difference depending on the energy requirements and cell type [15]. Mitochondria work together to optimize the mitochondrial pool in the cell via two processes termed fission and fusion. Fission is the process of splitting one mitochondrion into multiple mitochondria, which is integral for maintaining mitochondrial numbers during cell division and optimizing energy production when the metabolic capacity is high [16]. Additionally, mitochondria with aberrant cristae structure or abnormal mitochondrial morphology undergo “uneven” fission, where a smaller daughter mitochondrion will partition damaged components to be degraded via a process termed mitophagy [17]. Mitophagy is an organelle-specific form of autophagy, where damaged mitochondria are tagged via signaling proteins, engulfed by an autophagosome, and degraded in the lysosome. One of the most studied signaling pathways is PINK1/Parkin-mediated mitophagy. Briefly, under normal physiological conditions, the N-terminus of PINK1 is imported to the IMM via intermembrane transporters and mitochondrial membrane potential. Cleavage of the N-terminus allows for clearance of PINK1 from the OMM, maintaining low levels of PINK1 when mitochondria are metabolically healthy [18]. However, when mitochondria become damaged, depolarization of the IMM inhibits N-terminus import, resulting in an accumulation of PINK1 in the OMM causing autophosphorylation. Autophosphorylation triggers a signaling cascade and activation of Parkin, which tags the mitochondria for degradation via lysosomes [19]. Mitophagy is an integral process in mitochondrial dynamics, and dysfunctional mitophagy has been linked to a variety of cardiovascular diseases [20]. Fusion, on the other hand, is the joining of two or more mitochondria into one larger mitochondrion. Fusion is predicated to maintain cellular respiration when carbon substrates are low and may help to establish mitochondrial networks in certain cell types. Like fission, fusion is associated with the quality control of damaged mitochondria. As previously discussed, pathogenic variants in the mtDNA can be inherited through the maternal germline and/or acquired with age. Literature suggests that mitochondria with deficient OXPHOS due to heteroplasmy can fuse together to improve overall function, seemingly diluting the population of pathogenic mtDNA in the mitochondrion [21,22].

## 2. Mitochondrial Function in the Heart

As stated previously, the number and structure of mitochondria in distinct cell types can vary depending on energy demands. The myocardium is one of the most energy-demanding organs in the body. Despite necessitating such a large metabolic input, the myocardium has little capacity to store energy in the form of fat or glycogen. Therefore, continuous ATP production by OXPHOS in the mitochondria is integral for myocardium health and function. It is not surprising then that relative to other cells types in the body, the cardiac myocytes are rich in mitochondria, comprising 25–30% of the total cell volume [23]. Cardiac metabolism is unique from other cell types in that the majority of ATP produced in the cell is through ß-oxidation of fatty acids (~70%) [24]. However, glucose oxidation via glycolysis and the subsequent production of acetyl-CoA from pyruvate is an important fuel source for ATP production. Moreover, lactate, ketones, and amino acids can also be utilized by the cardiac cells and play an important role in cardiac metabolism [25,26]. While flexible in its metabolic capacity, the balance of substrate metabolism is seemingly important, as a shift in metabolic profile has been linked to decreased cardiovascular efficacy and multiple cardiovascular diseases [27].

### 2.1. Mitochondrial Organization in the Cardiomyocytes

Unlike other cell types, mitochondria are highly organized in the cardiomyocytes and can be categorized into three distinct populations based upon location: perinuclear, subsarcolemmal, and intermyofibrillar mitochondria [28]. Interestingly, beyond subcellular location, these mitochondrial subpopulations have a distinct structure and functions within the myocardium. ATP production, the response to external stimuli, and protein expression vary between populations. For example, the ATP generated by subsarcolemmal mitochondria are utilized for sarcolemma transport, while the ATP generated by intermyofibrillar mitochondria are thought to be used for muscle contraction. Interestingly, a functional distinction of these subgroups is calcium ion (Ca^2+^) handling, which is integral for cardiomyocyte health and function as described below [28].

### 2.2. Ca^2+^ Homeostasis in the Cardiomyocytes

It is well established that proper calcium homeostasis is integral to the function of the cardiomyocytes. In the cytoplasm, Ca^2+^ is essential for the contraction of the myocardium. An increase in intracellular Ca^2+^ is compulsory during systole, which promotes the interaction of actin and myosin. Opposing this action is the decrease in intracellular Ca^2+^ during diastole, causing relaxation of the myocardium [29]. In addition to its role in cardiac muscle contraction, Ca^2+^ is necessary in the mitochondrial matrix for the function of multiple enzymes during oxidative respiration. Research has demonstrated that increased levels of matrix Ca^2+^ upregulates the activity of pyruvate dehydrogenase and complex V, potentially through the modulation of the mitochondrial membrane potential, leading to amplified ATP production [30,31,32]. Therefore, during higher energy demand during contraction, ATP production simultaneously increases, supplementing the required energy needs of the cell. While the sarcoplasmic reticulum is the main reservoir of Ca^2+^ in the cell, mitochondria also have the capacity to sequester a large amount of Ca^2+^ [33]. More recently, it was appreciated that the rapid uptake of intracellular Ca^2+^ can occur via the mitochondria calcium uniporter (MCU) [34]. Contrasting this is the efflux of Ca^2+^ through the Na^+^-Ca^2+^-Li^+^ exchanger [35]. When the mitochondrial uptake of Ca^2+^ exceeds its retention capacity, mitochondrial Ca^2+^ overload can occur, leading to cell apoptosis and death, triggered by the opening of the mitochondrial permeability transition pore (mPTP) [36]. Calcium dysregulation has been implicated in several cardiovascular disease pathologies, highlighting the importance of proper Ca^2+^ homeostasis in the cardiomyocytes.

### 2.3. Iron Regulation and the Mitochondria

While the production of ATP is the primary function of the mitochondria, the mitochondria play an integral role in multiple other metabolic pathways essential for cell survival. Recently, the role of mitochondria in iron metabolism has gained substantial attention due to the description of a novel iron-regulated cell death termed ferroptosis [37]. Iron is required for multiple cellular processes including oxygen transport, DNA replication, and electron transport in the ETC [38]. Despite the importance of iron metabolites for cellular processes, the oxidation of ferrous ions (Fe^2+^) to ferric ions (Fe^3+^) via the Fenton reaction is a substantial source of ROS within the cell. As such, the opposing action of antioxidant systems, namely glutathione peroxidases, is required to maintain physiological levels of ROS [39]. The primary mechanism to which iron is transported into the cardiomyocyte is bound to transferrin. Transferrin binds to the transferrin receptor 1, signalling clathrin-dependent endocytosis. That being stated, multiple modes of transport have been shown to contribute to iron import in the cardiomyocyte in both homeostatic and pathogenic conditions [40]. Contrary, a single transporter, ferroportin, is responsible for iron export in the cardiomyocyte. Therefore, the disruption of ferroportin can easily result in iron overload, as observed in doxorubicin-induced cardiomyopathy [41]. Once endocytosed, Fe^3+^ is reduced to Fe^2+^, and transported into the cytosol where it can enter the cytosolic labile iron pool or be stored for future use within ferritin [42]. A significant amount of cellular iron stores are transported and utilized in the mitochondria [43]. Iron trafficking to the mitochondria has yet to be fully elucidated; however, a solvent-occluded mechanism has been hypothesized in cardiac cells [44]. Transport across the OMM is speculative at this point, but voltage-gated ion channels are suspected to play a role [45]. Conversely, iron transport across the IMM is better characterized with the solute carriers mitoferrin 1 and mitoferrin 2, thought to be the primary importers of iron into the mitochondrial matrix [46]. Interestingly, inhibition of the MCU impedes iron transport into the matrix, suggesting that calcium import is linked with iron homeostasis [47]. Within the mitochondria, iron has three functions: storage via mitochondrial ferritin, the generation of iron sulfur clusters, and heme synthesis. Both heme and iron sulfur clusters have several roles in the cardiomyocyte. Importantly, within the mitochondria, iron sulfur clusters and heme act as cofactors for varying complexes within the ETC [48]. Taken together, it is of no surprise that the dysfunction of iron homeostasis has disastrous consequences for the cardiomyocyte. Mitochondrial iron overload has been shown to result in a decreased production of ATP, a reduced complex activity, and eventual cell apoptosis in vitro [49]. On the other hand, iron depletion has been demonstrated to impair ATP production and the contractability of cardiomyocytes in vitro, demonstrating the need for the tight regulation of iron stores in the cell [50]. The importance of iron homeostasis in the cardiomyocyte is further supported by the discovery of ferroptosis. While many signalling pathways have been implicated in the ferroptotic cell, hallmark features include the suppression of antioxidant systems, increased iron-mediated ROS production, membrane lipid peroxidation, and a shift in lipid metabolism; ultimately leading to the destruction of cell membrane integrity. Morphologically, ferroptotic cells demonstrate small, fragmented mitochondria with abnormal cristae structure and OMM disruption [51]. Ferroptosis has been implicated in the progression of several cardiovascular diseases, indicating the need for further exploration of iron metabolism in the heart [40].

## 3. Mitochondrial Cardiomyopathy

Mitochondrial disease typically presents as a syndromic, multiorgan disease, preferentially impacting systems with high energy demands. Common symptoms may include muscle weakness, exercise intolerance, fatigue, vision problems, hearing impairment, cardiomyopathy, and/or nervous system abnormalities. Disease caused by mitochondrial dysfunction can be categorized into primary and secondary mitochondrial disease. Primary mitochondrial disease (PMD) is caused by pathogenic variants in the mtDNA or nDNA. PMD can be further be classified based upon the inheritance pattern and location of the disease-causing variant. Over 1000+ genes in the nDNA have been associated with mitochondrial disease. These variants can be inherited in autosomal dominant, autosomal recessive, and X-linked inheritance patterns. As described previously, the mtDNA is exclusively inherited from the maternal germline and can exist in homoplasmic or heteroplasmic states. When an mtDNA population harbors a pathogenic variant, homoplasmy or heteroplasmy can result in disease. Secondary mitochondrial disease refers to mitochondrial dysfunction secondary to another disease state. One such example is nutrient malabsorption in the gastrointestinal tract resulting in deficiencies of essential vitamins and cofactors needed for normal mitochondrial function [52]. Secondary mitochondrial disease can also be the result of another genetic etiology such as an inherited muscular dystrophy or an inborn error of metabolism [53]. For the purposes of this review, we will be focusing on PMD caused by pathogenic variants in mtDNA. One of the most common manifestations of mitochondrial cardiomyopathy is non-sarcomeric-related hypertrophic cardiomyopathy (nsHCM), which presents as a phenocopy of sarcomeric-related hypertrophic cardiomyopathy but is considered a distinct form of cardiovascular disease. nsHCM describes the abnormal thickening of the walls of the left ventricle, which can lead to ventricular arrhythmias and atrial fibrillation. HCM is diagnosed in children after an adjustment for body surface area with a Z-score >2 above the standard deviation of the mean. In adults, a maximal wall thickness ≥15 mm is considered diagnostic [54]. Following nsHCM, dilated cardiomyopathy (DCM) is a common cardiovascular manifestation in mitochondrial cardiomyopathy. DCM is characterized by the enlargement of one or both of the ventricles with an ejection fraction <50%, in the absence of a secondary disease [55]. Rarely, HCM progressing to DCM has been reported, and is often thought of to be a sign of end stage cardiovascular disease [56].

### 3.1. Syndromic mtDNA-Related PMD and Cardiomyopathy

Cardiomyopathy is a frequent finding in many forms of mtDNA-related PMD. Pathogenic variants in the mtDNA have been associated with decreased complex activities, reduced ATP production, abnormal mitochondrial dynamics, calcium dysregulation, and/or iron accumulation (to be discussed in Section 4) [57]. Therefore, it is of no revelation that individuals with pathogenic variants in the mtDNA of the cardiomyocytes demonstrate myocardial dysfunction. Phenotypes alter depending on the location of the mtDNA variant and heteroplasmy burden in the cardiomyocytes. Complicating the possible spectrum of disease, mtDNA heteroplasmy levels are not consistent between organ tissues and heteroplasmy levels in the blood may not be representative of those in the myocardium. Moreover, recent evidence suggests that heteroplasmy burden is not always predictive of phenotype, and other factors, such as nuclear modulators, may impact the severity of disease [58]. This variance results in reduced penetrance and variable expressivity amongst affected individuals, even within the same family, making the manifestation of a cardiac phenotype difficult to predict in asymptomatic individuals. As cardiovascular manifestations can be a feature in most forms of mtDNA-related PMD, a baseline cardiovascular work-up should be completed after PMD diagnosis. A regular screening regimen should be considered in asymptomatic individuals. Below we will highlight several common mtDNA-related PMDs with known cardiac risks. Table 1, Table 2, Table 3, Table 4 and Table 5 list suspected pathogenic variants associated with varying mtDNA-related PMD. Variants were vetted by a review of four public databases utilized by the research and clinical community to aggregate genomic data: (1) Online Mendelian Inheritance in Man (OMIM), (2) ClinVar, (3) Genome Aggregation Database (gnomAD v3.1.2), and (4) MITOMap [59,60,61,62]. Mitochondrial DNA variants associated with OMIM phenotypes were listed, unless indicated to be associated with multifactorial inheritance or of uncertain/debated significance. OMIM IDs linking the variant to the associated publication described on the website are reported. A review of mitochondrial genome variants in the ClinVar database was conducted. ClinVar variants were indicated in the tables when classified as pathogenic or likely pathogenic by more than one contributor. Variants reported as pathogenic or likely pathogenic by a single contributor or of uncertain significance were not indicated. For the databases gnomAD v3.1.2 and MITOMap, variants were indicated if classified as pathogenic or likely pathogenic. Uncertain variants were not indicated.

#### 3.1.1. Mitochondrial Myopathy, Encephalopathy, Lactic Acidosis, and Stroke-Like Episodes (MELAS)

MELAS is a relatively common mtDNA-related PMD with an estimated prevalence of 3–18/100,000 in the general population [63,64]. MELAS can have a wide spectrum of presentations and is characterized by a varying combination of encephalopathy, stroke-like episodes, myopathy, exercise intolerance, diabetes, recurrent headaches, neuropathy, visual disturbance, and/or hearing loss. There are more than 13 mtDNA genes that have been associated with MELAS, the majority of which are located in tRNA genes (Table 1). The most common and well characterized causative variant of MELAS is the m.3243A > G variant in the *MT-TL1* gene, encoding mitochondrial leucine tRNA (tRNALeu^(UUR)^). Cardiovascular findings are reported in <25% of patients and cardiac phenotypes have been well characterized in the literature [65,66,67]. nsHCM is the most common cardiovascular finding in MELAS patients, with a high risk for congestive heart failure [68,69]. nsHCM progressing to DCM has been reported in long-term cardiac profiling [70]. Post-mortem analysis of MELAS heart tissue has been performed. Enzyme analysis suggest deficiencies in cytochrome C oxidase and succinate dehydrogenase, while histology shows paracrystalline and lipidic inclusions and enlarged mitochondria with abnormal cristae structure [71]. Like the m.13513G > A variant in MERRF syndrome (described below), the m.3243A > G variant has been associated with a Wolff–Parkinson–White (WPW)-like conduction pattern. Interestingly, case studies have reported a diagnosis of WPW syndrome several years prior to a diagnosis of MELAS, suggesting it could be an early indicator of the disease [72].

#### 3.1.2. Myoclonic Epilepsy with Ragged Red Fibers (MERRF)

MERRF is a rare mtDNA-related PMD that is most frequently caused by the m.8344A > G variant in the *MT-TK* gene, which codes for a mitochondrial lysine transfer RNA (tRNA^Lys^) (Table 2) [73]. Pathogenicity of the m.8344A > G variant is suspected to be due to aberrant post-transcriptional modifications leading abnormal translation in the mitochondria [74]. MERRF is quite rare, with an estimated prevalence of less than 0.5/100,000 individuals [75]. MERRF is described by myoclonus, generalized epilepsy, cerebellar ataxia, and mitochondrial myopathy. Characteristic red ragged fibers can be identified on muscle biopsy. While the main features of this condition are myoclonus and epilepsy, cardiac conduction defects have been described. Arrhythmias are common and may present with or without cardiomyopathy [76].

#### 3.1.3. Leigh Syndrome

Leigh syndrome is a well characterized and relatively common PMD, estimating to affect 1/32,000 individuals at birth [77]. Leigh syndrome is chiefly considered a neurodegenerative phenotype and is characterized by bilateral hyperintense signals in the basal ganglia on T2-weighted MRI [78]. Leigh syndrome frequently presents during infancy after the occurrence of an infection, illness, or a stressful event. Signs and symptoms include developmental regression, hypotonia, dystonia, ataxia, seizures, optic atrophy, nystagmus, and/or cardiovascular findings. Clinical findings can vary between individuals and milder phenotypes have been reported. Locus heterogeneity is observed in Leigh syndrome, with variants in both the mtDNA and nDNA associated with the manifestation of the disease [79]. mtDNA variants that have been reported in association with Leigh syndrome are listed in Table 3. It is appreciated that many of these mtDNA variants are in complex subunit coding genes, resulting in an abnormal complex function in the ETC. Common variants include the m.8993T > G/C and m.9176T > G/C variants in the *MT-ATP6* gene (complex V subunit), the m.10191 T > C in the *MT-ND3* gene (complex I subunit), and the m.13513 G > A variant in the *MT-ND5* gene (complex I subunit) (Table 3). The most common extraneurological manifestation of Leigh syndrome is cardiovascular disease. The prevalence of cardiovascular findings is estimated to affect 20–40% of patients, making cardiovascular monitoring important in this patient population [80]. nsHCM, DCM, and cardiac conduction abnormalities are commonly reported [81]. Sofou et al. published a case review of 96 Leigh syndrome patients, reporting that patients with mtDNA-related Leigh syndrome were more likely to develop nsHCM/DCM than those with nDNA-related Leigh syndrome. The onset of nsHCM/DCM in this cohort was typically later than those with nDNA-related Leigh syndrome [80]. While nsHCM/DCM can be seen in several mtDNA-related Leigh syndrome variants, the m.13513 G > A variant of Leigh syndrome is unique in its association with WPW syndrome, which can present as an isolated finding or alongside cardiomyopathy [82,83,84].

#### 3.1.4. Mitochondrial DNA Deletion Syndromes (MDDS)

MDDS are a rare PMD estimated to affect approximately 1/100,000 individuals at birth. MDDS describe a spectrum of three overlapping phenotypes: Kearns-Sayre syndrome, Pearson syndrome, and progressive external ophthalmoplegia [75]. Progressive external ophthalmoplegia is characterized by ophthalmoplegia, ptosis, and proximal limb weakness, while Pearson syndrome is a bone marrow failure syndrome distinguished by sideroblastic anemia and exocrine pancreas dysfunction. Finally, Kearns-Sayre syndrome presents with the classic triad of progressive external ophthalmoplegia, pigmentary degeneration of the retina, and cardiac conduction abnormality including heart block, prior to the age of 20 [85]. All three conditions are most frequently caused by large, heteroplasmic, mtDNA deletions and rearrangements, negatively impacting the expression of proteins required for OXPHOS and mitochondrial translation (Table 4). Poor prognosis and early death are common, with Pearson syndrome the most severe of the three clinical phenotypes [86]. Diagnosis for MDDS can be challenging, as selection against the mtDNA deletion and/or rearrangement can be observed in rapidly dividing tissues. Therefore, molecular genetic testing via a blood sample has a low sensitivity for detecting mtDNA deletion and/or rearrangements [87]. While the detection of low level heteroplasmy is improving with recent advances in sequencing technology, the gold standard for MDDS diagnosis is confirmation using DNA extracted from a muscle biopsy sample [88,89]. As stated previously, cardiac conduction abnormalities are a well characterized feature in Kearns-Sayre syndrome and often contribute to the mortality of the disease. Up to 20% of patients are reported to die from sudden cardiac death. Implantable cardioverter defibrillator placement as a preference over pacemaker placement and has been argued to be a standard of care for any Kearns-Sayre syndrome patients [90]. Syncope may be the first indication of cardiovascular dysfunction, followed by conduction defects [91,92]. Case reviews have suggested the average age of onset for cardiac complications is in the 2nd decade, with common conduction defects including right bundle branch block and fascicular blocks, eventually progressing to complete atrioventricular block [91,92,93]. Bradycardia-related polymorphic ventricular tachycardia is also frequently reported in Kearns-Sayre syndrome patients [92]. Cardiac muscle biopsy from Kearns-Sayre syndrome patients show a combination of enlarged mitochondria with abnormal cristae morphology, in addition to small vacuolized mitochondria [94].

### 3.2. Nonsyndromic mtDNA-Related Cardiomyopathy

While most mtDNA-related cardiomyopathy manifests in conjunction with other features of mitochondrial disease, non-syndromic, maternally inherited cardiomyopathy (MIC) has been reported (Table 5) [95,96]. Between this spectrum of mtDNA-related PMD and MIC are conditions designated cardiomyopathy plus, a term used to describe a PMD where cardiomyopathy is the predominant feature, with other milder associated findings of mitochondrial disease. Briefly, we will review several cardiac phenotypes that can be observed in MIC, a genetic assessment for MIC, and the discovery of novel mtDNA variants in MIC.

#### 3.2.1. Maternally Inherited Cardiomyopathy

By far the most frequent manifestation of MIC is nsHCM. As previously discussed, nsHCM is commonly observed in mtDNA-related PMD; however, non-syndromic cases have been reported [97,98]. As an example, the MELAS-associated m.3243A > G variant can present as MIC, having further implications for the affected individual and their biological family (to be discussed below). MIC-related nsHCM often presents with left ventricular systolic dysfunction, as opposed to left ventricular outflow tract obstruction that is more commonly associated with pathogenic variants in sarcomere-related HCM [99]. Following nsHCM, DCM is the next most common finding in MIC. DCM has been reported in both infantile and adult-onset MIC [100,101]. DCM may present as the primary cardiovascular manifestation or secondary to nsHCM. A less common phenotype reported in association with pathogenic mtDNA variants is left ventricular noncompaction cardiomyopathy (LVNC) [102]. LVNC is thought to be a failure of the cardiac compaction process during embryogenesis. Studies in MIC patient tissues with LVNC showed hypertrabeculation of the myocardium in association with abnormal mitochondrial morphology, including a disrupted and vacuolized cristae structure [103]. Isolated LVNC with accompanying hypertrophy has also been reported [104]. Lastly, another interesting, but rare, phenotype in association with MIC is histiocytoid cardiomyopathy. Histiocytoid cardiomyopathy is characterized by enlarged subendocardial nodules primarily composed of histiocyte-like myocytes and Purkinje fibers. Clinically, histiocyte cardiomyopathy presents with cardiomegaly, arrhythmia, and/or sudden death, with onset typically in infancy. It has been reported in several patients with the m.8344A > G-related MERRF variant, in addition to as an isolated finding in MIC [105,106,107].

#### 3.2.2. Clinical Assessment for MIC

The clinical assessment of MIC can be challenging due to the intrafamilial variance of the disease. While other influences such as nuclear modifiers and environmental factors likely contribute to the manifestation of MIC, the most prominent contributor to the appearance of MIC is heteroplasmy burden of the mtDNA variant in cardiac muscle. Due to the random segregation of mitochondria during gamete production, heteroplasmy levels within the cardiac muscle of members of the same family can vary. From there, consideration also needs to be made for the likely variance of heteroplasmy within different tissues of an affected proband. Heteroplasmy levels in the blood may not be reflective of the variant load in the cardiac muscle. Therefore, low blood heteroplasmy or a negative mtDNA genome analysis may be reconsidered using an alternative tissue type (e.g., urine, muscle). Moreover, the onset of MIC can range from infancy to adulthood, with some forms of infantile disease resulting in early death (Table 5) [97,108,109]. Taken together, this makes predicting phenotypes for both an affected individual and an asymptomatic carrier extremely challenging. As an example, we have previously discussed the common m.3243A > G variant as a cause of both severe multiorgan disease, in addition to being reported in individuals with isolated MIC [110,111]. This phenomenon highlights the need for proper counseling and education in this patient population, as women with MIC could go on to have a child with infantile multiorgan disease. Similarly, at-risk family members may not appreciate the risk for extracardiovascular manifestations of PMD if their signs and symptoms are different than other family members. In our clinical experience, a family history could be remarkable for subtle findings of mitochondrial disease over several generations (e.g., isolated hearing loss, diabetes, and/or stroke), which may go unappreciated until a severely affected family member is diagnosed with a PMD. This diagnosis may then identify several seemingly asymptomatic carriers, who have not established a proper cardiac screening protocol and may be at risk for MIC. With that being stated, in consideration of the overall genetic etiology of familial cardiomyopathy, mtDNA variants are a rarer causative factor for the disease [112,113]. As such, mtDNA analysis is not frequently included in panel testing. If suspicion is high, mtDNA sequencing should be considered as a second-tier analysis for isolated cardiomyopathy. A maternal inheritance pattern and/or a family history positive for other features suspicious of a mitochondrial etiology may indicate testing.

#### 3.2.3. Discovery and Classification of Novel mtDNA Variants in Association with MIC

**Figure 4:**
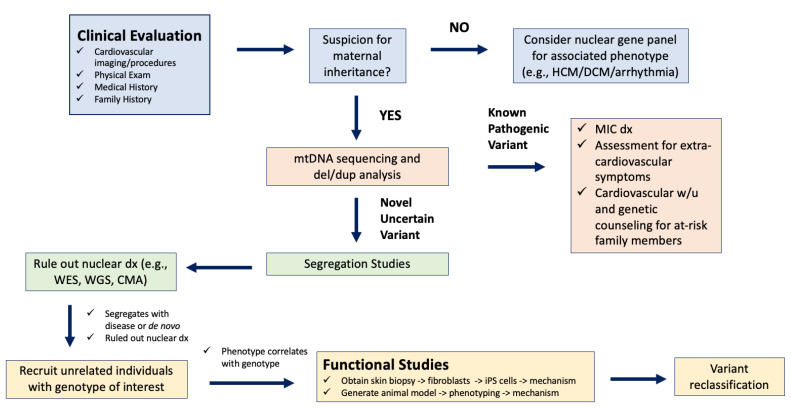
Research flow chart for novel mtDNA variant discovery. CMA, chromosomal microarray; del/dup, deletion/duplation; dx, diagnosis; DCM, dilated cardiomyopathy; HCM, hypertrophic cardiomyopathy; iPS cells, induced pluripotent stem cells; WES, whole exome sequencing; WGS, whole genome sequencing; w/u, work-up.

The analysis of mtDNA variants as a causative factor for MIC is complex. Formerly, mtDNA variant classifications were fraught with problems due to a lack of evidence standards, understanding of haplogroups, and the limited diversity of available population datasets [114,115]. While there is much room for improvements in this area, evidence standards and publicly available databases have made variant interpretation more reliable in the modern era [116,117]. Clinical laboratories use evidence frameworks to classify mtDNA variants into several categories. Typically, variants are classified as benign, likely benign, of uncertain significance, likely pathogenic, or pathogenic. These classifications are made using multiple lines of evidence, including an analysis of population frequency, haplogroup, variant properties, clinical correlation, and functional studies [116]. Our group, among many others, have made efforts to explore novel disease-causing variants in the mtDNA using clinical data and functional studies [97]. Our approach to the analysis of a novel mtDNA variant as a causative factor for MIC is summarized in Figure 4. Briefly, a genetic work-up begins by obtaining a detailed medical history and phenotypic information from the proband. This should be followed by obtaining a thorough family history and a minimum three-generation pedigree. When maternal inheritance is suspected, mtDNA analysis is recommended. The reporting of a novel mtDNA variant prompts consideration for further research. Supporting evidence can be acquired by the molecular testing of other family members. While this can be challenging due to the issues with heteroplasmy, generally speaking, if the variant is de novo (new in the proband), suspicion may be raised. Similarly, if the variant segregates with disease in the family, it is suggestive of variant pathogenicity. If found in the homoplasmic state in several unaffected family members, it would be more suggestive of a benign change. Next, even if suspicion is high, ruling out the possibility of another nuclear genetic etiology is required. Whole exome or whole genome sequencing may be considered. Following this assessment, the recruitment of unrelated individuals with the same genotype is recommended. Clinical correlation of the phenotype in multiple unrelated families is highly suggestive of the variant as a cause of disease. Lastly, functional experiments using patient-derived samples and/or available model systems can provide robust evidence that the mtDNA variant disrupts post-transcriptional modification, protein expression, mitochondrial function, and/or morphology, among other possibilities. Overall, clinicians and researchers play an important role in mtDNA variant analysis by sharing clinical phenotypes and producing functional data, aiding in the discovery of new disease-causing variants in this patient population.

### 3.3. mtDNA Variants as a Risk Factor for Multifactorial Cardiovascular Disease

While not the focus of this review, it is worthwhile to highlight the emerging evidence of mtDNA variants as a risk factor for multifactorial conditions such as atherosclerosis, coronary heart disease, myocardial infarction, and essential hypertension [118,119]. Multifactorial disease is thought to be influenced by a combination of both environmental and genetic factors, with higher population frequency variants in the mtDNA associated with an increased incidence of certain cardiovascular diseases. A 2018 study of 415 Polish individuals with atherosclerosis proposed a polygeneic model of mtDNA inheritance, finding that the number of mtDNA polymorphisms was correlated to the manifestation of the disease [119]. Similarly, studies of mitochondrial variants associated with familial hypertension and coronary heart disease demonstrate an increased prevalence of variants in mitochondrial tRNA coding genes. Functional studies in this patient population report defects in tRNA structure leading to decreased energy production and subsequent ROS [120,121]. Further research into the utility of mtDNA sequencing for polygenetic and multifactorial cardiovascular disease may help guide identifying at-risk individuals and the development of therapeutics in this patient population.

## 4. Mechanisms of Pathophysiology

While the association of mtDNA pathogenic variants and cardiomyopathy is clear, the mode of pathogenicity has yet to be fully elucidated. The mechanism of disease likely involves multiple mechanisms impacting the overall efficacy and health of the mitochondrial cardiomyocyte population. The study of mtDNA-related PMD in vivo has been challenging due to the lack of proper model systems [122]. However, evidence derived from in vitro studies and animal models of nuclear gene-related PMD have provided multiple insights. Identifying the underlying mechanism of mtDNA-related cardiomyopathy is of upmost importance for the development of targeted therapeutics in this patient population. Below we will propose possible mechanisms of pathophysiology in mtDNA-related cardiomyopathy.

### 4.1. Insufficient Energy Metabolism in the Cardiomyocyte

The most straightforward hypothesis of mtDNA-related cardiomyopathy is insufficient ATP production due to suboptimal OXPHOS in the cardiomyocytes. Due to the composition of the mtDNA, the majority of mtDNA pathogenic variants are in tRNA coding genes (Figure 2). While heterogenous, overall, pathogenic variants in tRNA coding genes can impair transcriptional termination, post-transcriptional modifications, and the translation of proteins from the mtDNA [121,123]. The downstream effect being lowered mitochondrial fitness and respiration capacity. Multiple functional studies have shown decreased complex activity and OXPHOS in patients with mtDNA-related cardiomyopathy [124,125]. Similarly, a transmitochondrial cytoplasmic hybrid (cybrid) of m.3243A > G has demonstrated that increasing heteroplasmy levels significantly upregulates both mtDNA and nDNA genes involved in OXPHOS and the TCA cycle, suggesting deficient energy production in these cells [126]. Energy deficiency has also been reported in DCM patients via the non-invasive measurement of the myocardial phosphocreatine/ATP ratio. Reduced ratios were associated with a higher likelihood of mortality related to cardiovascular disease [127]. Interestingly, studies using m.3243A > G cybrid cell lines reported that increasing heteroplasmy levels results in a shift in metabolism from glucose oxidation, to the uncoupling of glycolysis from oxidative phosphorylation resulting in the cytosolic metabolism of pyruvate [126,128]. While it is difficult to extrapolate this data to mtDNA-related cardiomyopathy, a metabolic shift in glucose and fatty acid metabolism and an abnormal lactate/pyruvate ratio has been consistently reported in the literature in association with failing hearts [25]. As the myocardium has little capacity for energy storage, an emergent shift to ATP production independent from the ETC is a logical adaptive mechanism to compensate for temporary energy exhaustion. However, long term uncoupling of glycolysis from glucose oxidation results in the accumulation of lactate, a well-known marker of mitochondrial dysfunction, and is associated with early signs of heart failure [129]. Due to the energic requirements of the cardiac muscle, a likely initial pathology for the development of mtDNA-related cardiomyopathy is insufficient ATP production via suboptimal OXPHOS.

### 4.2. Abnormal ROS Homeostasis

Mitochondrial genome variants have a well-established association with increased ROS production [130]. Mechanisms for how mtDNA variants increase ROS production have been proposed. The mtDNA codes for 13 complex subunits and pathogenic variants in these genes that can result in a misfolded or structurally altered protein product [131]. All ETC complexes are multiprotein structures and structurally abnormal subunits can impact complex assembly and subsequent function. Complex I and III are suspected to be the highest contributors to ROS production in the healthy cell; therefore, impaired complex function led to a “leaky” complex and an increased ROS production. Under normal conditions, ROS has important signaling roles in the cardiomyocyte. ROS signaling has been implicated with cardiomyocyte cell differentiation, cell signaling, vascular smooth muscle relaxation, redox modifications of Ca^2+^ channels, and contractibility in the cardiac muscle [132,133,134]. However, under pathological conditions ROS signaling in the cardiac muscle can become detrimental. A positive feedback loop of ROS production was first reported by Zorov et al. who described a phenomena termed ROS-induced ROS release [135]. During oxidative stress in the mitochondria, two IMM channels termed the mPTP and inner membrane anion channel, can open allowing for the passage of ions between the intermembrane space and the mitochondrial matrix. The opening of the mPTP and/or inner membrane anion channel has several consequences for the mitochondria. Under normal physiological circumstances, temporary uncoupling causes depolarization of the IMM, allowing the mitochondria to release excess ROS into the intermembrane space to relieve oxidative stress. During pathological conditions, a prolonged opening of the inner membrane anion channel or mPTP can trigger cell death and ROS-induced ROS release. More specifically, sustained decoupling of the membrane potential inhibits ATP production via the ETC and causes mitochondrial swelling due to the influx of small molecules into the matrix. This uncoupling eventually leads to mitochondrial damage and the release of mitochondrial contents into the cytosol [136]. ROS release from one mitochondrion into the cytoplasm acts as a signal of oxidative stress to neighboring mitochondria, who in turn release ROS into the cytosol creating a “burst” of ROS in the cardiomyocyte [137]. Indeed, ROS-induced ROS release has been shown to contribute to reperfusion injury and heart failure for individuals experiencing myocardial infarction due to dysfunctional mPTP function [138]. Besides ROS, the abnormal homeostasis of mitochondrial Ca^2+^ (to be discussed below) can trigger both ROS production and mPTP opening, creating an unfavorable synergistic effect in the cell [139]. Moreover, it has been demonstrated that ROS-induced damage to the mtDNA itself may play a role in cardiac dysfunction through the oxidation of guanine into the DNA lesion 7,8-dihydro-8-oxoguanine (8-oxo-dG), suggesting a possible feedback mechanism by which increased ROS may contribute to ever accelerating mtDNA damage in cardiomyopathy [140]. It is therefore likely that mtDNA genome variants, especially those that impact complex functions, cause an increased production of ROS leading to acquired mtDNA mutations exacerbating ETC dysfunction, depolarization of the mitochondrial membrane, and cell stress, contributing to the manifestation of mtDNA-related cardiomyopathy.

### 4.3. Altered Mitochondrial Dynamics

As previously described, mitochondrial dynamics are essential to maintain a healthy population of mitochondria. It was formerly suspected that mitochondria in adult cardiomyocytes were hypodynamic, with little to no fission and fusion in order to maintain the integrity of the ridged structure of the myofibrils [141]. However, nDNA fission-deficient or fusion-deficient conditional cardiac mouse models have suggested otherwise, with mice developing DCM with accompanying cell death and impaired respiratory fitness, respectively [142]. This work demonstrates the necessity of proper mitochondrial dynamics and the adverse impact of abnormal mitochondrial morphology in the cardiomyocyte. A consistent feature of mtDNA-related cardiomyopathy on electron microscopy is large, structurally abnormal mitochondria with atypical cristae structure, and an increased number of vacuolized mitochondria [143,144,145]. This has similarly been shown in cybrids of m.3243A > G of varying heteroplasmy, with increased mutation burden associated with elongated, fragmented, and swollen mitochondria. While this finding could be associated with uncoupling and swelling of the mitochondrial matrix as described above in ROS-induced ROS release, it is possible that this morphology is indicative of sustained mitochondrial fusion, an established compensatory response to insufficient OXPHOS caused by mtDNA genome variants [21,22]. Additionally, sustained fusion could also indicate impaired fission and mitophagy in the cell. While fusion has been hypothesized to dilute deleterious mtDNA molecules, improving the fitness of the resulting mitochondria, an opposing theory can be modelled for fission. By the nature of fission, multiple fission events (or network fragmentation) lessen the copy number of mtDNA in the resulting mitochondrion. Theoretically, this can result in daughter mitochondrion with higher heteroplasmy levels, leading to mitochondrial dysfunction, membrane depolarization, and destruction via PINK1/Parkin-mediated mitophagy. This process would eliminate a pool of deleterious mtDNA from the mitochondrial network. Indeed, upregulation of Parkin has shown to shift heteroplasmy levels in cybrid cells, restoring complex activities and ETC function [146]. Interestingly, mitochondrial network fragmentation has also been demonstrated to be protective of cardiomyocyte function during increased energy demand under normal physiological conditions, suggesting a role of mitochondrial fragmentation in the cardiomyocyte [147]. While a compelling strategy to reestablish homeostasis in the mitochondrial network, in mtDNA-related PMD, theoretically, most mitochondria likely harbor a population of deleterious mtDNA. Fragmentation could lead to mass destruction of multiple mitochondria, lowering energy production to deleterious levels. Moreover, dysfunctional mitochondria could trigger ROS-mediated ROS release and eventual cell death. Interestingly, mitophagy and lowered expression of PINK1/Parkin is consistently reported in various cardiovascular diseases [20]. Taken together, one could speculate that fusion is biased in mtDNA-related PMD, protecting the cell from unwanted consequences of network fragmentation while maintaining mitochondrial function through the dilution of heteroplasmy levels. Unfortunately, as a highly organized subcellular organelle in the myocardium, an alteration to the organization of the mitochondrial network may contribute to the disruption of sarcomere structure. Indeed, case reports have demonstrated these large abnormally structured mitochondria in conjunction with disrupted sarcomere structure on electron microscopy staining [148]. As previously described, the disruption of sarcomere structure is a well-established cause of HCM and DCM, with multiple nDNA pathogenic variants in sarcomere genes resulting in familial cardiomyopathy [54]. Often described as a phenocopy of HCM, it is reasonable to suspect that mitochondrial cardiomyopathy is disruptive of sarcomere structure, possibly secondary to abnormal mitochondrial dynamics.

### 4.4. Calcium Dysregulation in the Heart

While the interplay of cytosolic and mitochondrial Ca^2+^ in calcium regulation, sequestration, and signaling are still under scrutiny, the dysfunction of Ca^2+^ homoeostasis in the cardiomyocyte is a well characterized feature of heart failure. Under normal physiological conditions, an increase in matrix Ca^2+^ is associated with activation of the TCA cycle and subsequent ATP production [30,31,32]. An influx of Ca^2+^ into the matrix can occur via the MCU but can also be transferred from the sarcoplasmic reticulum. While typically cytosolic and mitochondrial Ca^2+^ are partitioned to participate in alternative signaling pathways in the cardiomyocyte, a connection between the two compartments has been characterized and termed mitochondrial-associated membranes. Mitochondrial-associated membranes facilitate the transfer of lipids and Ca^2+^ between the two organelles, and are generated by the fission and fusion proteins that mediate mitochondrial dynamics [149]. During times of high energy demand, increased mitochondrial-associated membranes allow for an influx of Ca^2+^ from the sarcoplasmic reticulum into the matrix and subsequent TCA stimulus [150]. While this mechanism may be adaptive under temporary stress, similar to ROS production, when critical levels of matrix Ca^2+^ are present in the mitochondria, opening of the mPTP occurs, leading to membrane depolarization, possible ROS-induced ROS release, and cell damage [151]. Indeed, the knockout of fission and fusion proteins demonstrate abnormal Ca^2+^ recycling and dysfunctional contractility in association with abnormal Ca^2+^ levels [152]. In addition to matrix Ca^2+^, cytosolic Ca^2+^ are a well characterized contributor to the pathophysiology of cardiomyopathy. As previously discussed, mtDNA-related cardiomyopathy is frequently associated with increased ROS production. ROS modify several regulators of cytosolic Ca^2+^, raising the level of Ca^2+^ in the cytosol, triggering a large influx of Ca^2+^ via ryanodine receptor 2 [153,154]. Compounding, ROS directly modify ryanodine receptor 2 via cysteine residues, which has been shown to generate a significant leak of sarcoplasmic Ca^2+^, further impacting cardiomyocyte contractability [155]. Additional exploration of Ca^2+^ signaling in mtDNA-related cardiomyopathy may provide integral insights on the role of Ca^2+^ regulation in the pathophysiology of disease.

### 4.5. Iron Overload in the Mitochondria

An emerging area of research is the role of iron metabolism in the pathophysiology of mitochondrial cardiomyopathy. Iron overload associated with cardiomyopathy has been documented in multiple nDNA-related diseases, frequently associated with disorders of heme or iron sulfur metabolism [156,157]. This observation suggests that iron homeostasis is, at least, partially maintained by the utilization of the trace metal during the production of iron sulfur clusters and heme in the mitochondria. While speculative, it could be theorized that when these pathways are disrupted, the influx of iron surpasses the mitochondria’s ability to utilize the trace element, resulting in mitochondrial iron overload. Besides defects in iron metabolism pathways, overload can be observed in selected phenotypes associated with mtDNA-related PMD, posing the question of how deleterious mtDNA variants result in iron accumulation in the mitochondria [158]. A straightforward hypothesis mimics the theory stated above, that energy depletion in mtDNA-related PMD results in mitochondria that are unable to utilize the iron pool at an efficient rate, leading to accumulation. However, another interesting consideration is iron overload secondary to abnormal Ca^2+^ regulation. Studies in isolated murine cardiac mitochondria demonstrate that an exposure to Fe^2+^ and Fe^3+^ in vitro results in increased ROS and membrane depolarization, mimicking conditions of iron overload. When these same mitochondria are introduced to a pharmacological inhibition of the MCU, the deleterious effects of iron overload are not observed, suggesting that iron uptake is either directly or indirectly regulated by the MCU [159]. As both Ca^2+^ and Fe^2+^ are required cofactors for complexes in the ETC, it is plausible that during times of increased energy demand, both ions are rapidly imported into the matrix for use by the ETC complexes via the MCU. However, as an influx of Fe^2+^ can contribute to ROS accumulation and lipid peroxidation in the mitochondria, prolonged influx as a response to energy depletion could trigger ferroptosis. Interestingly, in vitro studies in murine fibroblast cells, demonstrate that inactivation of glutathione peroxidase 4 leads to lipid peroxidation, followed by an influx of Ca^2+^ and end stage ferroptosis, suggesting an interplay between Ca^2+^ and Fe^2+^ in ferroptotic signalling [160]. Given the important role of Ca^2+^ in the cardiomyocyte, characterizing the interplay between iron and calcium signalling may lead to novel therapeutics to combat mtDNA-related cardiomyopathy.

## 5. Conclusions

Taken together, mtDNA-related cardiomyopathy is a heterogenous condition that can be syndromic or non-syndromic in nature. It is caused by pathogenic variants in the mitochondrial genome that result in a dysfunctional gene product, including mitochondrial tRNA, rRNA, or ETC complex subunits. The presentation is highly influenced by heteroplasmy levels, which can differ from tissue to tissue and therefore demonstrate intrafamilial variance. Mitochondrial genome variants likely cause the progression of mtDNA-related cardiomyopathy via multiple compounding mechanisms. Our proposed hypothesis of possible pathophysiology is as follows: (1) Pathogenic mtDNA variants negatively impact ATP production and ETC activity, resulting in energy depletion. (2) Due to the high metabolic requirement of cardiac muscle, alterative metabolic pathways are activated to compensate for energy demands, subsequently uncoupling glycolysis from glucose oxidation and increasing lactate production. (3) To “reset” OXPHOS in damaged mitochondria, fusion occurs to dilute the level of heteroplasmy in the mitochondria, resulting in enlarged and hyperfused mitochondria, impacting sarcomere structure and mitochondrial network organization, further exacerbating energy depletion. (4) To help combat cellular stress from energy depletion, increased mitochondrial-associated membrane formation occurs, allowing for Ca^2+^ transfer from the sarcoplasmic reticulum to the mitochondrial matrix. Further, opening of the MCU allows for supplementary import of Ca^2+^, in addition to Fe^2+^, into the matrix. (5) As mitochondrial metabolism is dysfunctional, Fe^2+^ cannot be utilized at an appropriate rate relative to Fe^2+^ influx, and accumulated Fe^2+^ is oxidized via the Fenton reaction, leading to ROS production and lipid peroxidation. (6) Concurrently, leaky complex activity contributes to the accumulation of ROS in the matrix. The ultimate result of all these factors is that the threshold is reached to open the mPTP, causing depolarization of the mitochondrial membrane and mitochondrial damage. ROS release into the cytosol has three possible results: the modification of calcium channels leading to an increase in cytosolic calcium and dysfunctional cardiomyocyte contractibility, lipid peroxidation triggering ferroptosis, and possible ROS-induced ROS release signaling to neighboring mitochondria. Together, these mechanisms contribute to the pathophysiology of mtDNA-related cardiomyopathy. While speculative, further examination of interacting mechanisms of mtDNA-related cardiomyopathy will allow for the development of effective therapeutics in this patient population.

## Figures and Tables

**Table 1:** Known pathogenic mitochondrial genome variants associated with mitochondrial encephalopathy, lactic acidosis, and stroke-like episodes (MELAS) ^†^.

Gene	Gene Product	mtDNA Variants *	OMIM ID #	Ref.	Cardiovascular Findings Associated with Phenotype **
*MT-ND1*	Complex I subunit	m.3697G > A m.3946G > A m.3949T > C	516000.0012 516000.0013 516000.0014	[A] [A]	nsHCM DCM LVNC WPW Cardiac conduction abnormality
*MT-ND5*	Complex I subunit	m.12770A > G m.13042G > A m.13045A > C m.13084A > T **m.13513G > A**	516005.0004 516005.0008 516005.0005 516005.0006 516005.0007	[A][C] [A][C] [A] [A] [A][C]	
*MT-ND6*	Complex I subunit	m.14453G > A	516006.0005	[A][C]	
*MT-TC*	tRNA ^Cys^	m.5814A > G	590020.0001		
*MT-TF*	tRNA ^Phe^	m.583G > A	590070.0001	[A][C]	
*MT-TH*	tRNA ^His^	m.12147G > A m.12158A > G	590040.0003	[A][C] [C]	
*MT-TK*	tRNA ^Lys^	m.8316T > C m.8356T > C	590060.0002	[C] [A][B][C]	
*MT-TL1*	tRNA ^Leu(UUR)^	**m.3243A > G** **m.3252A > G** m.3256C > T m.3258T > C **m.3271T > C** m. T > C	590050.0001 590050.0005 590050.0003 590050.0002	[A][B][C] [A] [A][C] [C] [A][C] [C]	
*MT-TM*	tRNA ^Met^	m.4450G > A		[C]	
*MT-TQ*	tRNA ^Gln^	m.4332G > A	590030.0003	[A][B][C]	
*MT-TS1*	tRNA ^Ser(UCN)^	m.7512T > C	590080.0001	[A]	
*MT-TS2*	tRNA ^Ser(AGY)^	m.12207G > A	590085.0002	[A][C]	
*MT-TW*	tRNA ^Trp^	m.5541C > T		[C]	

^†^ DCM, dilated cardiomyopathy; nsHCM, non-sarcomeric-related hypertrophic cardiomyopathy; LVNC, left ventricular noncompaction; OMIM ID #, Online Mendelian Inheritance in Man ID number; Ref., reference; WPW, Wolff–Parkinson–White syndrome; [A] Reported as pathogenic or likely pathogenic in the database ClinVar by more than one contributor. Pathogenic variants submitted by a single contributor and uncertain variants were not indicated; [B] Reported as pathogenic or likely pathogenic in the database gnomAD v3.1.2. Uncertain variants were not indicated; [C] Reported as pathogenic or likely pathogenic in the data base MITOMap. Uncertain variants were not indicated. * Common variants are in bold. ** Cardiac manifestations commonly associated with MELAS. MELAS-associated variants may present with varying cardiovascular phenotype depending on the heteroplasmy levels and location of the variant.

**Table 2:** Known pathogenic mtDNA variants associated with myoclonic epilepsy with ragged red fibers (MERRF) ^†^.

Gene	Gene Product	mtDNA Variants *	OMIM ID #	Ref.	Cardiovascular Findings Associated with Phenotype **
*MT-ND5*	Complex I subunit	m.13042G > A	516005.0008	[A][C]	nsHCM DCM Cardiac conduction abnormality
*MT-TF*	tRNA ^Phe^	m.611G > A	590070.0002	[A]	
*MT-TH*	tRNA ^His^	m.12147G > A	590040.0003	[A][C]	
*MT-TK*	tRNA ^Lys^	**m.8344A > G** **m.8356T > C** **m.8361G > A** **m.8363G > A**	590060.0001 590060.0002 590060.0007 590060.0003	[A][B][C] [A][B][C] [A] [A][B][C]	
*MT-TL1*	tRNA ^Leu(UUR)^	m.3243A > G m.3256C > T	590050.0001 590050.0003	[A][B][C] [A][C]	
*MT-TP*	tRNA ^Pro^	m.15967G > A	590075.0003	[A][C]	
*MT-TS1*	tRNA ^Ser(UCN)^	m.7512T > C	590080.0001	[A]	
*MT-TS2*	tRNA ^Ser(AGY)^	m.12207G > A	590085.0002	[A][C]	

^†^ DCM, dilated cardiomyopathy; nsHCM, non-sarcomeric-related hypertrophic cardiomyopathy; OMIM ID #, Online Mendelian Inheritance in Man ID number; Ref., reference; [A] Reported as pathogenic or likely pathogenic in the database ClinVar by more than one contributor. Pathogenic variants submitted by a single contributor and uncertain variants were not indicated; [B] Reported as pathogenic or likely pathogenic in the database gnomAD v3.1.2. Uncertain variants were not indicated; [C] Reported as pathogenic or likely pathogenic in the database MITOMap. Uncertain variants were not indicated. * Common variants are in bold. ** Cardiac manifestations commonly associated with MERRF. MERRF-associated variants may present with varying cardiovascular phenotype depending on the heteroplasmy levels and location of the variant.

**Table 3:** Known pathogenic mtDNA variants associated with Leigh syndrome ^†^.

Gene	Gene Product	mtDNA Variants *	OMIM ID #	Ref.	Cardiovascular Findings Associated with Phenotype **
*MT-ND1*	Complex I subunit	m.3460G > A m.3481G > A m.3890G > A m.3946G > A	516000.0001 516000.0013	[A][B][C] [A] [A] [A]	nsHCM DCM WPW Cardiac conduction abnormality
*MT-ND2*	Complex I subunit	m.4640C > A m.4681T > C	516001.0003 516001.0006	[A]	
*MT-ND3*	Complex I subunit	m.10158T > C **m.10191T > C** m.10197G > A	516002.0003 516002.0001 516002.0004	[A][C] [A][C] [A][C]	
*MT-ND4*	Complex I subunit	m.11777C > A	516003.004	[A][C]	
*MT-ND5*	Complex I subunit	m.12706T > C m.13042G > A m.13063G > A m.13084A > T **m.13513G > A** m.13514A > G	516005.0003 516005.0008 516005.0006 516005.0007	[A][C] [A][C] [A][B] [A] [A][C] [A][C]	
*MT-ND6*	Complex I subunit	m.14459G > A m.14484T > C m.14487T > C	516006.0002 516006.0001 516006.0007	[A][B][C] [A][B][C] [A][C]	
*MT-CO3*	Complex IV subunit	m.9478T > C m.9537dupC	516050.0005	[B] [A]	
*MT-ATP6*	Complex V subunit	m.8783G > A m.8839G > C m.8851T > C **m.8993T > G** **m.8993T > C** m.9035T > C **m.9176T > G** **m.9176T > C** m.9185T > C m.9191T > C	516060.0001 516060.0002 516060.0011 516060.0005 516060.0008	[A][B] [A] [A][B] [A][C] [A][B][C] [A][B][C] [A][C] [A][B][C] [A][C] [A][C]	
*MT-TK*	tRNA ^Lys^	m.8363G > A	590060.0003	[A][B][C]	
*MT-TM*	tRNA ^Met^	m.4450G > A		[C]	
*MT-TS2*	tRNA ^Ser(AGY)^	m.12258C > T m.12264C > T		[A][C] [A][C]	
*MT-TV*	tRNA ^Val^	m.1624C > T m.1630A > G m.1659T > C	590105.0002	[A][B] [A][C] [A]	
*MT-TW*	tRNA ^Trp^	m.5523T > G m.5537insT m.5540G > A m.5543T > C	590095.0002	[C] [A] [A] [A]	

^†^ DCM, dilated cardiomyopathy; nsHCM, non-sarcomeric-related hypertrophic cardiomyopathy; OMIM ID #, Online Mendelian Inheritance in Man ID number; Ref., reference; WPW, Wolff–Parkinson–White syndrome; [A] Reported as pathogenic or likely pathogenic in the database ClinVar by more than one contributor. Pathogenic variants submitted by a single contributor and uncertain variants were not indicated; [B] Reported as pathogenic or likely pathogenic in the database gnomAD v3.1.2. Uncertain variants were not indicated; [C] Reported as pathogenic or likely pathogenic in the data base MITOMap. Uncertain variants were not indicated. * Common variants are in bold. ** Cardiac manifestations commonly associated with Leigh syndrome. Leigh syndrome-associated variants may present with varying cardiovascular phenotypes depending on the heteroplasmy levels and location of the variant.

**Table 4:** Known pathogenic mtDNA variants associated with mitochondrial DNA deletion syndromes (MDDS) ^†^.

Gene	Gene Product	mtDNA Variants	OMIM ID #	Ref.	Cardiovascular Findings Associated with Phenotype *
*MT-TL1*	tRNA ^Leu (UUR)^	m.3249G > A m.3255G > A	590050.0011	[A][C]	nsHCM DCM Cardiac conduction abnormality
*MT-TL2*	tRNA ^Leu (CUN)^	m.12315G > A	590055.0001	[A][C]	
**Deletion/** **Duplications**	Various	Multiple reported		[A][B][C]	

^†^ DCM, dilated cardiomyopathy; nsHCM, non-sarcomeric-related hypertrophic cardiomyopathy; OMIM ID #, Online Mendelian Inheritance in Man ID number; Ref., reference; [A] Reported as pathogenic or likely pathogenic in the database ClinVar by more than one contributor. Pathogenic variants submitted by a single contributor and uncertain variants were not indicated; [B] Reported as pathogenic or likely pathogenic in the database gnomAD v3.1.2. Uncertain variants were not indicated; [C] Reported as pathogenic or likely pathogenic in the data base MITOMap. Uncertain variants were not indicated. * Cardiac manifestations commonly associated with MDDS. MDDS-associated variants may present with varying cardiovascular phenotype depending on the heteroplasmy levels and location of the variant.

**Table 5:** Known mtDNA variants associated with maternally inherited cardiomyopathy (MIC) ^†^.

Condition	Gene	Gene Product	mtDNA Variants	OMIM ID #	Ref.	Other Findings
**nsHCM**	*MT-CYB*	Complex III subunit	m.14849T > C	516020.0012		Septo-optic dysplasia, retinitis pigmentosa
	*MT-ATP6*	Complex V subunit	m.8528T > C	516060.0010	[A]	Infantile
	*MT-ATP8*	Complex V subunit	m.8528T > C m.8529G > A	516070.0003 516070.0002	[A] [A]	Neuropathy
	*MT-TG*	tRNA^Gly^	m.9997T > C	590035.0001	[C]	Myopathy Exercise intolerance
	*MT-TI*	tRNA ^Ile^	m.4295A > G m.4300A > G m.4317A > G	590045.0003 590045.0006 590045.0001	[A][C]	Infantile, SNHL Infantile
	*MT-TK*	tRNA^Lys^	m.8363G > A	590060.0003	[A][B][C]	SNHL
	*MT-TL1*	tRNA ^Leu (UUR)^	m.3243A > G m.3260A > G m.3303C > T	590050.0001 590050.0007 590050.0004	[A][B][C] [A][C] [A][C]	MELAS overlap Skeletal myopathy Skeletal myopathy
	*MT-TV*	tRNA ^Val^	m.1624C > T m.1644G > A	590105.0002	[A][B] [A][B][C]	Leigh overlap MELAS overlap
	*MT-TW*	tRNA ^Trp^	m.5545C > T	590095.0001		Multisystem
**DCM**	*MT-TH*	tRNA^His^	m.12192G > A	590040.0001		LHON overlap
	*MT-TI*	tRNA ^Ile^	m.4269A > G m.4300A > G m.4317A > G m.4322dupC	590045.0002 590045.0006 590045.0001	[A][C] [A][C] [C]	Multisystem Infantile
	*MT-TL1*	tRNA ^Leu (UUR)^	m.3243A > G	590050.0001	[A][B][C]	MELAS overlap
	*MT-TL2*	tRNA ^Leu (CUN)^	m.12297T > C	590055.0003		
	*MT-TR*	tRNA ^Arg^	m.10415T > C		[C]	
	*MT-TY*	tRNA^Try^	m.5843A > G	590100.004		Focal segmental glomerulosclerosis
**LVNC**	*MT-TL1*	tRNA ^Leu (UUR)^	m.3243A > G	590050.0001	[A][B][C]	MELAS overlap
	*MT-TV*	tRNA ^Val^	m.1612C > T		[C]	
**Histiocytoid cardiomyopathy**	*MT-CYB*	Complex III subunit	m.15498G > A	516020.0011	[C]	Leigh overlap
	*MT-TK*	tRNA ^Lys^	m.8344A > G	590060.0001	[A][B][C]	

^†^ DCM, dilated cardiomyopathy; nsHCM, non-sarcomeric-related hypertrophic cardiomyopathy; LHON, Leber hereditary optic neuropathy; LVNC, left ventricular noncompaction cardiomyopathy; MELAS, mitochondrial encephalopathy, lactic acidosis, and stroke-like episodes; OMIM ID #, Online Mendelian Inheritance in Man ID number; Ref., reference; SNHL, sensorineural hearing loss; [A] Reported as pathogenic or likely pathogenic in the database ClinVar by more than one contributor. Pathogenic variants submitted by a single contributor and uncertain variants were not indicated; [B] Reported as pathogenic or likely pathogenic in the database gnomAD v3.1.2. Uncertain variants were not indicated; [C], Reported as pathogenic or likely pathogenic in the data base MITOMap. Uncertain variants were not indicated.

## Data Availability

Not applicable.
